# Liver extracellular vesicles in health and disease

**DOI:** 10.3402/jev.v1i0.18825

**Published:** 2012-07-11

**Authors:** Felix Royo, Juan M. Falcon-Perez

**Affiliations:** 1 Metabolomics Unit, CIC bioGUNE, CIBERehd, Technology Park of Bizkaia, Derio, Bizkaia, Spain; 2 IKERBASQUE, Basque Foundation for Science, Bilbao, Spain

**Keywords:** extracellular vesicles, exosomes, microvesicles, microparticles, liver, hepatocyte

## Abstract

Extracellular vesicles (EVs) play an important role in cell-to-cell communication. Although there are different kinds of vesicles, each with their own secretion and capture biology, all of them carry a cargo of proteins, lipids, metabolites and nucleic acids. They act as vehicles for exchange of biological materials and signals and are involved in the regulation of various physiological processes. Liver is an essential organ containing different cell populations fulfilling various functions, which need to be strictly controlled and coordinated. There are a few articles showing the role of liver-derived EVs. On the basis of them, we present here a hypothesis of the implication of such vesicles in the physiology of the liver. Different liver cell types, including hepatocytes, cholangiocytes and stellate cells, secrete and capture EVs and interact with them. Liver injury changes the abundance and cargo of EVs; these changes are likely to be important for the outcome of stress response. Although a substantial effort has been put into the characterization of EVs in isolated populations, it is only recently that some more comprehensive information has begun to appear. In this article, we hypothesize about the role of EVs in liver microenvironment and their possible function using published data from both hepatic and non-hepatic systems.

Liver is a multifunctional organ involved in important metabolic functions, synthesis of blood components and storage of lipids and retinol (1). It is also responsible for the disposal of bacterial products taken up from the gut and detoxification of xenobiotics and drugs. To accomplish these tasks, the liver employs not just hepatocytes, but also other non-parenchymal immune and non-immune cells. The resident liver tissue macrophages (Kupffer cells), natural killer (NK) cells, T cells and B cells are all the members of the hepatic immune system. These cell types strictly regulate the immune system in liver and are important mediators in inflammation (2). Among non-immune cells, the hepatic stellate cells, also known as Ito cells, are involved in angiogenesis (3) and fibrosis processes. The parenchyma of liver consists mostly of hepatocytes, which are polarized cells with important roles in detoxification, production and clearance of blood components, and in the formation of bile.

All these cellular populations, with their diverse physiological processes, have to be strictly coordinated to perform their functions properly. Recently, the role of secreted extracellular vesicles in the exchange of proteins, nucleic acids and lipids has awakened some considerable interest in the field of intercellular signalling (4). Two main groups of secreted vesicles have been identified: endosome-derived vesicles named exosomes and plasma membrane-shed vesicles called ectosomes or microparticles (5). Here we refer to both as extracellular vesicles (EVs), following the nomenclature adopted by the International Society of Extracellular Vesicles (http://www.isevmeeting.org). However, we also provide the type of EVs, if the information is available.

There have been just few studies focusing on the role of circulating liver-derived EVs, or the possible effects that non-hepatic EVs have on liver. Supported by those published articles, and extrapolating information obtained from non-liver systems, we offer here a collection of possible scenarios where EVs may be important for liver functionality.

Several reports (6–8) have identified and characterized the role of EVs in intercellular communications; 2 fundamental mechanisms have been proposed. First, the vesicle membranes can interact with receptors of the targeted cell, triggering signal pathways. Some of the EV membrane proteins cannot be identified on the surface of the donor cell, and activate a signalling path different to that employed during direct cell contact (9). Second, EVs can be internalized (4), and their cargo, including proteins and various types of nucleic acids (such as mRNA and miRNA), can be then released (7, 10).

Remarkably, the composition of EVs depends on the particular cell state (11); their extracellular localization makes them an ideal research target in the field of biomarker discovery (12, 13). Identification of molecular markers for early detection and prognosis of liver conditions is one of the main tasks in hepatology. Currently, diagnosis of liver diseases relies mainly on histological examination of liver biopsies. However, liver biopsy is invasive and its practical applications are limited by sampling errors, low diagnostic accuracy and hazard to the patient (14, 15). Biopsy is often used late in the disease progress and the lesions might be irreversible in many cases. These concerns have been fuelling the search for novel non-invasive markers for the diseases associated with liver injury, such as steatosis, hepatitis, fibrosis, apoptosis, necrosis and cancer cell proliferation. The isolation and characterization of liver-derived EVs in blood samples could shed some new light on various liver physiological processes (5). Our aim here is to hypothesize about the role of EVs and their components in the hepatic environment, both in health and disease (Figure 1 and Table I).

**Fig. 1:**
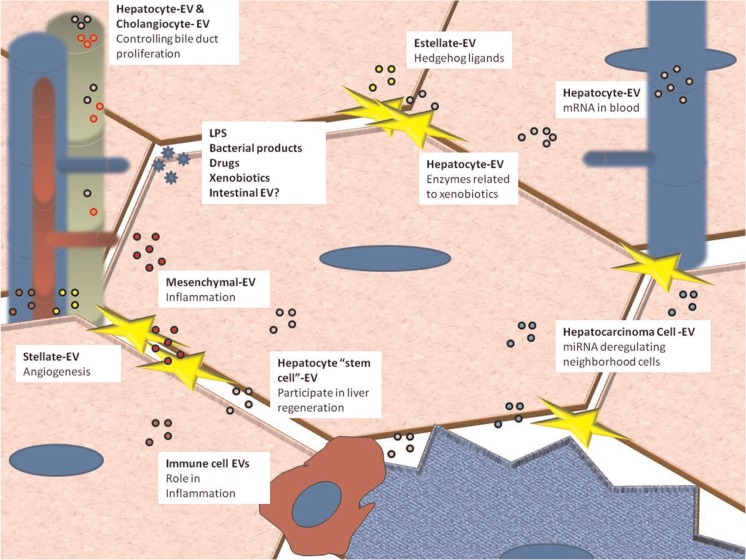
Liver cells are able to release and capture EVs. The small circles represent EVs released from different cells. Green corresponds to cholangiocytes, red to blood-circulating EVs released from other tissues, yellow to stellate cells, violet to tumoral cells, pink to hepatocytes and brown to immune cells. For references, see text and Table I.

**Table I:** EV-secreting liver cell types; different studies show the properties and relevance of the EVs’ cargo for each cell type

Cell type secreting EVs	Cargo analysed	Relevance	Reference
Cholangiocytes	Protein	Hedgehog ligands	(45)
Cholangiocytes	miRNA-15a	Modulation ERK phosphorylation	(47)
Hepatic stellate cells	Protein	Hedgehog ligands	(45)
Hepatocellular cancer cell	miRNA	Silence of TAK1	(65)
Hepatocytes	Protein	Drug detoxification	(16)
Hepatocytes	RNA	Marker of liver damage	(24)
Leucocytes	Protein	Inflammation	(61)
Pluripotent liver cells	RNA	Regeneration	(23)

## EVs related to drug metabolism

Previously, we have reported that non-tumoral liver-derived cell lines such as MLP29 and primary cultured hepatocytes are able to secrete EVs (16). A comprehensive proteomic study of EVs released by primary hepatocytes has identified several members of cytochrome P450, uridinediphosphate-glucuronosyl-transferase (UGT) and glutathione S-transferase (GST) protein families (17). The proteomics data were validated on fractions obtained by employing a sucrose density gradient thereby demonstrating the vesicle-associated nature of the proteins. The presence of hepatocyte-derived EVs in the bloodstream is indirectly supported by the fact that EVs purified from plasma contain the enzyme carbamoyl phosphate synthetase 1, which is highly expressed in the liver and to a lesser extent in the small intestine tissue (17, 18).

Our proteomic analysis of hepatocyte-derived EVs indicates that these vesicles contain several members of the P450, UGT and GST drug-metabolizing protein families. If hepatocyte-derived EVs would be released to the blood stream, it can be hypothesized that EVs might be involved in detoxification of drugs and endogenous toxic substances (13). Supporting this hypothesis, it has been already described that tumour cells employ EV-mediated transport to develop and confer chemotherapy resistance. Hence, P-glycoprotein is a transmembrane protein involved in multidrug resistance that is transferred via EVs from drug-resistant cancer cells to drug-sensitive cells (19). Moreover, cisplatin-resistant cell lines release this anti-tumoral drug and its transporters by using an EV-dependent mechanism, transferring the survival ability to acceptor cells (20–22).

## Circulating liver-specific mRNA associated with EVs

Hepatocyte-derived EVs contain mRNA (23), and liver-specific mRNAs associated with EVs are present in the bloodstream (24). Blood samples obtained after galactosamine-induced liver damage in animal models show increased levels of liver-specific mRNAs associated with EVs and cell debris. For detecting liver damage, amplification and quantification of these nucleic acids in blood samples have proved to be more sensitive than traditional transaminase activity quantification (24). As the activity of transaminase enzymes increases in some other conditions, such as muscle injury (25, 26), mRNA quantification methods are also more specific (24). Assessment of circulating albumin mRNA levels as a measure of liver damage has been used in a chronic and mild fibrosis model, and has also been shown to be more sensitive than transaminase activity measurements (27). However, that study has not determined whether the albumin mRNA was associated with EVs.

In general, the role of bloodstream mRNAs is still unclear. Since the first descriptions of mRNA associated to EVs, functional transfer of EV-associated RNA to the acceptor cells has been shown (10). However, to prove unequivocally that the mRNA present in the EVs finally is translated into a functional protein by the acceptor cells, it has required plasmid overexpression of green fluorescence protein (GFP) mRNA in EV-producing cells and resulting GFP expression in the acceptor cell (28). Furthermore, it has been demonstrated that bone marrow cells co-cultured with liver cells, separated by a cell-impermeable membrane, express mRNA for albumin (29, 30). These data suggest that EVs both deliver mRNA to bone marrow cells and mediate the transcription of tissue-specific mRNA although more investigations in this area are clearly needed.

Although it has been suggested that the role of EVs is to protect RNA from RNase activity, the available data are controversial. The first description of RNA in exosomes has shown that mRNA is protected against RNase activity (10). However, RNase treatment of EVs preparations has also been employed as a proof that the EV-dependent phenotype is mediated by an RNA component (23, 28). One possible interpretation of these results is that different EVs confer different degree of protection. However, it is also plausible that certain RNAs might be located outside the vesicle lumen as associated membrane complexes. It is important to note that these studies employed different doses of RNase and very different times of treatment; it is also not clear whether fresh or frozen samples were used.

## EV pathway for virus propagation

Hepatitis C virus (HCV) is a major cause of chronic liver disease, with about 170 million people infected worldwide (31). The HCV uses its envelope glycoproteins E1 and E2 to bind specific receptors such as CD81, claudin-1 and the class B member I scavenger receptor in the host cells (32, 33). HCV entry involves an additional clathrin-mediated post-internalization step and delivery into early endosomes (34). However, it is not clear how the assembled HCV virion is released from the cytoplasm. Extensive research on human immunodeficiency virus (HIV) has shown that the ESCRT (endosomal sorting complex required for transport) machinery plays a role in virion assembly (35). This machinery is also fundamental in the synthesis and sorting of exosomal cargo (36, 37). In HCV-infected patients, viral RNA is associated with EVs in plasma (38). In addition, the HRS protein, involved in the autophagic pathway (39), is required for exosome secretion, and plays an important role in HCV release (38–40). Improving our knowledge of EVs’ involvement in viral propagation could be useful in the development of new therapies against viral infections.

## EVs in regeneration and differentiation

Liver is involved in detoxification of xenobiotics and noxious endobiotics, and therefore continuously exposed to injuries; tissue regeneration is an important defensive mechanism maintaining the viability of this organ. In acute liver injury, hepatic regeneration is not unlike a physiological wound-healing process, bringing about transient and reversible changes in the extracellular matrix of the organ and in the proliferative capacity of the hepatocytes (41, 42).

When administered *in vivo*, EVs derived from a subpopulation of human pluripotent resident liver cells accelerate the morphological and functional recovery of liver in hepatectomized rats (23). This effect is lost when EVs are treated with RNase, suggesting that RNA is involved in the process (23). Stem cells also use a horizontal transfer of mRNA to redirect the behaviour of differentiated cells (28, 43), and EVs participate in the cross-talk between stem and differentiated cells (44).

Protein cargo also plays an important role in response to EVs. Cholangiocytes and myofibroblastic hepatic stellate cells release EVs containing active hedgehog ligands in response to platelet-derived growth factor (45). In the acceptor cells, these EVs activate hedgehog signals that might stimulate angiogenesis (45).

## EVs and cholangiocytes

Recently, it has been reported that cholangiocytes can secrete EVs (45). Moreover, transmission electron microscopy observations show that EVs present in the bile duct interact with the primary cilia of cholangiocytes (46). In experimental models of bile duct-ligated rats, biliary exosomes have been detected *in vivo* (45).

Biliary EVs secreted by cholangiocytes take part in the inactivation of ERK kinase signalling (47). This pathway is associated with the inhibition of cholangiocyte proliferation. Interestingly, a decrease in the ratio of phospho-ERK to total ERK correlates with the activation of miR-15a transcription in cholangiocytes treated with EVs. Inhibition of this miRNA also enhances cholangiocyte proliferation (48). However, the specific composition of cholangiocyte-derived EVs that mediate this effect remains unknown.

## EVs in liver inflammation

In developed countries, non-alcoholic fatty liver disease is emerging as a major global liver disorder with the background of an increasing prevalence of obesity and type 2 diabetes (49). Non-alcoholic fatty liver disorder encompasses a spectrum of diseases from simple steatosis through steatohepatitis to fibrosis, and ultimately cirrhosis and hepatocellular carcinoma. Hepatocellular carcinoma is the fifth most common cancer worldwide and the third most common cause of cancer mortality (50).

The role of EVs in inflammation is ambiguous because EVs can elicit either inflammation (51), or immunosuppressive effects (52, 53). In shock patients, circulating EVs increase oxidative stress in liver (54). Notably, hepatocyte death induced by chronic oxidative stress and inflammation triggers a potent regenerative response in attempt to restore the hepatic parenchyma (41). It has been shown that certain EVs can act as antigen vehicle (55). In cases of liver injury there is a large number of circulating EVs (24, 56) that contain liver-specific enzymes (57). Therefore, the possible involvement of EVs in autoimmune hepatitis should not be rejected (17). Aberrant activation of innate immune signalling may trigger “harmful inflammation” that contributes to sepsis, chronic inflammation, autoimmune diseases, tissue and organ injuries, fibrosis and carcinogenesis (2). Disruption of the intestinal epithelial barrier results in a leaky gut, which causes bacterial translocation and the appearance of bacterial products, such as lipopolysaccharides and toxins, in the liver (2, 58, 59). A number of obesity-related factors have been proposed as stimuli that activate the Toll-like receptors (TLR) pathway (60). In a fatty-liver rat model caused by high fatty diet, EVs isolated from peripheral blood were able to activate CD11b+ cells with subsequent homing to and accumulation of the cells in the liver (61).

## EVs’ miRNA and cancer

Tumoral cells undergo deregulation of miRNAs expression (62) and are known to release miRNAs associated with EVs (12, 63, 64). An exhaustive catalogue of miRNAs present in EVs released by hepatic cancer cells has pointed towards TAK1 protein as one of the central targets of tumour-derived EV miRNA (65). TAK1 is an essential inhibitor of hepatocarcinogenesis, and the lack of TAK1 *in vivo* is associated with the spontaneous development of hepatocellular carcinoma as a result of aberrant responses to inflammatory and stress signalling (66, 67). TAK1 can also have a direct effect on cancer progression through repression of the telomerase reverse transcriptase gene (68). The aberrant expression of specific miRNAs in tumour cells and the ability of miRNAs to modulate multiple oncogenic or tumour suppressor genes make them well suited for such a role (65).

## The objective is the network

Figure 1 shows a comprehensive summary of the possible roles of EVs secreted by liver cells. However, EVs derived from certain cell types have not been described; there is no data on hepatic sinusoidal cells, which play an important role in the cross-talk and paracrine regulation of lymphocytes and hepatocytes (69, 70). Further research will be required for *in vivo* isolation and characterization of other liver-derived EVs.

The appropriate regulation of the components present in any microenvironment is crucial to the stress response. As we have mentioned before, obesity causes leucocyte accumulation (71). Leucocyte EVs might trigger a response that activates immune cells which in turn rise the levels of interleukins (61). Oxidative stress induces hepatocyte damage (41) and the amount of liver-derived EVs probably increases (24, 56). EVs derived from liver resident pluripotent cells could induce regenerative signals (23). Hypothetically, liver-derived EVs might be captured by antigen-presenting cells (55) in the bone marrow and perhaps trigger an autoimmune response causing severe liver damage and inflammation. Steatohepatitis and fibrosis favour hepatocarcinogenesis and, in a final step of our hypothetical model, tumoural cells would release miRNA-containing EVs that deregulate the neighbouring cells (65, 66).

Maintaining appropriate signal balance may be crucial for the resolution of liver injuries. In a multicellular environment, EVs from different cell types, carrying different cargos, might interact with parenchymal cells. It is likely that liver dysfunction affects the composition of EVs’ cargo, making those vesicles invaluable in the damage assessment using plasma analysis. From another point of view, the ability of liver cells to uptake circulating EVs makes them a silver bullet in gene and miRNA therapy. The challenge is to decode the EVs’ network and find the tools to control it.
